# Pediatric Rhabdomyosarcomas of the Genitourinary Tract

**DOI:** 10.3390/cancers15102864

**Published:** 2023-05-22

**Authors:** Jennifer T. Castle, Brittany E. Levy, Derek B. Allison, David A. Rodeberg, Eric J. Rellinger

**Affiliations:** 1 Department of Surgery, Markey Cancer Center, University of Kentucky, Lexington, KY 40536, USA; jennifer.castle@uky.edu; 2 Department of Surgery, University of Kentucky, Lexington, KY 40536, USA; brittany.levy@uky.edu; 3 Department of Pathology and Laboratory Medicine, Department of Urology, Markey Cancer Center, University of Kentucky, Lexington, KY 40536, USA; derek.allison@uky.edu; 4 Department of Surgery, Department of Pediatric Surgery, University of Kentucky, Lexington, KY 40536, USA; david.rodeberg@uky.edu; 5 Department of Surgery, Department of Pediatric Surgery, Markey Cancer Center, University of Kentucky, Lexington, KY 40536, USA

**Keywords:** genitourinary rhabdomyosarcoma, pediatric soft tissue sarcomas, bladder and prostate rhabdomyosarcoma, paratesticular rhabdomyosarcoma, female genitourinary rhabdomyosarcoma

## Abstract

**Simple Summary:**

Rhabdomyosarcomas are soft tissue tumors more commonly found in children than adults. The diagnosis and management of these tumors continues to change as our knowledge of how they behave and how they respond to previously used treatments has improved. We treat these tumors differently based on where they develop. Rhabdomyosarcomas of the genitourinary tract are the second most common, arising from the bladder, prostate, vagina/uterus, and paratesticular regions. Advancements in treatment options, including chemotherapy and radiation, have led to improved outcomes and have allowed, in many instances, for less drastic surgical resections. In this review, we discuss how to approach rhabdomyosarcomas in general, with an emphasis on the presentation, diagnosis, treatment, and outcomes of those arising from the different organs in the genitourinary tract.

**Abstract:**

Rhabdomyosarcoma (RMS) is the most common soft tissue sarcoma in the pediatric and adolescent population, with 350 new cases diagnosed each year. While they can develop anywhere in the body, the genitourinary tract is the second most common primary location for an RMS to develop. Overall survival has improved through the increased use of protocols and multidisciplinary approaches. However, the guidelines for management continue to change as systemic and radiation therapeutics advance. Given the relative rarity of this disease compared to other non-solid childhood malignancies, healthcare providers not directly managing RMS may not be familiar with their presentation and updated management. This review aims to provide foundational knowledge of the management of RMSs with an emphasis on specific management paradigms for those arising from the genitourinary tract. The genitourinary tract is the second most common location for an RMS to develop but varies greatly in symptomology and survival depending on the organ of origin. As the clinical understanding of these tumors advances, treatment paradigms have evolved. Herein, we describe the breadth of presentations for genitourinary RMSs with diagnostic and treatment management considerations, incorporating the most recently available guidelines and societal consensus recommendations.

## 1. Introduction

Rhabdomyosarcoma (RMS) is the most common soft tissue sarcoma in pediatric and adolescent populations [1]. There are approximately 350 new diagnoses of RMSs each year, with a bimodal distribution demonstrating the highest incidence in children under four and adolescents [1,2]. RMSs are mesenchymal in origin and, thus, can develop nearly anywhere in the body, although, most commonly, the trunk, extremities, and head/neck regions are affected. Approximately 18–22% develop in the genitourinary tract (i.e., bladder, kidney, prostate, paratesticular, vulva, vagina, cervix, and uterus), making the GU tract the second most common site for RMSs [3,4]. RMSs in the GU tract (GU RMSs) are conventionally classified as bladder/prostate (BP-RMSs) or non-bladder/prostate RMSs. Female non-bladder/prostate (FGU-RMS) primaries arise from the vagina, vulva, and/or uterus, while male non-bladder/prostate RMSs are paratesticular. While there are genetic syndromes with an increased risk of developing an RMS (detailed later), environmental exposures (e.g., prenatal x-ray exposure, incomplete immunizations, and increasing maternal age) have been demonstrated in some studies to increase the risk of developing an RMS, but these interactions are understudied, often contradictory across studies, and are not specific to the development of RMSs in the GU tract [5]. Overall, protocolized, multidisciplinary approaches to the management of RMSs have resulted in improved outcomes (~70% overall survival). RMSs have heterogeneous outcomes based upon a variety of features, including histology, genetic features, stage of disease, and primary site. Current investigations have primarily been focused on identifying cases with unfavorable features in need of more intensive or novel treatment strategies and those with favorable prognostic factors that may suffer less morbidity from the de-escalation of therapy. Given their close proximity to adjacent organs in the pelvis, treatment of GU RMSs can be challenging with secondary lifelong sequelae. This review is aimed to provide knowledge of the management of RMSs with an emphasis on those arising from the genitourinary tract by merging the organ-specific paradigms and clinical considerations into a single consolidated review.

## 2. Histology

According to the 2020 World Health Organization update on the classification of soft tissue tumors, RMSs are divided into four histologic subtypes–embryonal, alveolar, spindle cell/sclerosing, and pleomorphic [6]. An embryonal RMS (ERMS) generally carries a favorable prognosis and consists of oval-spindle-shaped cells with morphologic features resembling fetal skeletal muscles. It accounts for 80% of all GU RMSs. Botryoid is an embryonal variant and is most common in hollow cavities, including the vagina and bladder. Histological evaluation of botryoid subtypes has demonstrated dense clusters of polypoid-appearing cells abutting an epithelial surface. Gross examination of these lesions resembles a “bunch of grapes”. Spindle cell subtypes resemble smooth muscles and occur most commonly in the paratesticular region, head and neck, orbit, and extremities [7,8]. The alveolar (ARMS) subtype is comprised of round cells in nests surrounded by fibrovascular septae, giving it a pseudo-alveolar or lung-like architecture. This morphologic subtype is a poor prognostic factor associated with higher rates of metastasis [7]. A pleomorphic RMS is extremely rare in children and most commonly originates in the extremities. Similar to pleomorphic RMSs, seen in adults, children can present with tumors containing varying levels of anaplasia. Anaplasia is most common in embryonal RMSs, favorable sites of origin, and lower-risk disease. While diffuse anaplasia had previously been reported as an indicator of worse outcomes compared to focal anaplasia, a multivariate analysis of patients from five different Children’s Oncology Group (COG) trials did not find the presence of focal or diffuse anaplasia as a significant indicator of clinical outcome [9].

## 3. Tumor Biology

Molecular features have usurped histologic classification in the risk stratification of RMSs in the most recent COG trials [10,11]. The majority of ARMSs (80%) feature a genetic translocation joining *PAX3* or *PAX7* DNA-binding domains fused with the transactivation domain of *FOXO1* [12]. This gene fusion creates a transcription factor that supports malignant transformation by enhancing cell proliferation, survival, and invasion [13]. *PAX3* and *PAX7*-*FOXO1* fusions are associated with lower event-free survival, but only *PAX3*-*FOXO1* fusions are associated with significantly worse survival when compared to fusion-negative ARMS and ERMS [12]. Twenty percent of ARMSs lack *PAX3* or *PAX7*/FOXO1 translocations. Despite their histologic appearance, these fusion-negative ARMSs (FN-ARMSs) behave more similarly to ERMSs with comparable overall and event-free survival [12,14]. The majority of tumors arising in the GU tract are fusion-negative with embryonal histology. With advances in genetic sequencing, the genetic landscape of RMSs continues to grow, with now over 20 different fusion genes identified with varying levels of known clinical significance [15].

The majority of pediatric RMSs occur sporadically, but a subset are linked to germline mutations that predispose to developing an RMS. Familial syndromes with increased risk of developing RMSs include Li-Fraumeni syndrome, Neurofibromatosis type 1, Costello syndrome, *DICER1* syndrome, Beckwith-Wiedemann syndrome, and Noonan syndrome, which, respectively, feature mutations in *TP53*, *NF1*, *HRAS*, *DICER1*, imprinting centers on chromosome 11, and various genes involved in the RAS pathway [5,15]. There are over 30 genetic mutations, both sporadic and familial, identified in RMS patients with pathogenic or likely pathogenic predilections [15,16]. Familial syndromes can have a site-specific predilection for RMS development. For example, *DICER1* mutations are most commonly found in the female GU tract [16]. In contrast, *TP53* mutations in RMSs have a much broader anatomic distribution, including the extremities, female GU tract, and head and neck, but are less frequently seen in bladder/prostate and paratesticular regions [16,17]. When evaluating patients with an RMS, it is crucial to consider genetic screening as patients with specific genetic mutational profiles may benefit from targeted therapeutics and those with syndromic disease will require the appropriate surveillance. Additionally, having a genetic counselor as a member of the multidisciplinary care team is beneficial in addressing familial counseling.

## 4. RMS Stage

RMSs are staged pretreatment based on physical examination and imaging studies using the TNM staging system. Staging of RMSs takes into consideration the primary tumor site, size, and invasiveness, along with the lymph node spread and presence or absence of distant metastatic disease. Non-bladder/prostate GU RMSs have less aggressive clinical courses and are frequently staged lower than BP-RMSs [11]. A size greater than 5 cm and extension/fixation to surrounding structures contribute to staging primary tumors. In addition, the nodal stage comprises N0 (no regional lymph node involvement), N1 (regional lymph nodes clinically involved defined as measuring >1 cm on CT/MRI), and Nx (regional lymph node status unknown). Lastly, the metastatic stage comprises M0 (no distant metastasis) and M1 (distant metastasis present) [11].

## 5. Clinical Groups

RMSs are clinically grouped following biopsy or resection with pathologic analysis prior to the initiation of adjuvant therapy, such as chemotherapy. The clinical group is used to determine the need for radiation therapy (RT) and contributes to the overall risk stratification in guiding the chemotherapeutic protocol selection. These clinical groupings were first categorized in 1972 by the Intergroup Rhabdomyosarcoma Study (IRS) group [18]. Group I tumors are localized to the site of origin and completely resected without lymph node involvement. Group II describes tumors that are localized to the site of origin but with microscopic residual disease after resection and/or evidence of regional nodal spread. Group III describes tumors that are incompletely resected with gross residual disease, which includes tumors that are only biopsied (excluding complete excisional biopsy). Lastly, Group IV describes tumors with distant metastases [11].

## 6. Risk Stratification

Genetic features, along with the staging and clinical grouping, contribute to modern risk stratification, which guides chemotherapy intensity and duration. A summary of RMS risk stratification is highlighted in Table 1. Fusion-positive tumors (*PAX3/7-FOXO1* mutation) are associated with significantly worse outcomes, making these tumors higher risk [12]. In addition, *TP53* and *MYOD1* mutations are also considered in the risk stratification, given the evidence of worse outcomes associated with these mutations independent of fusion status [15]. Historically, RMSs were classified into low, intermediate, and high-risk groups. However, recently there has been the emergence of a very low-risk (VLR) group, which is currently being used in stratifying patients for clinical trials [10,11].

**Figure 1:**
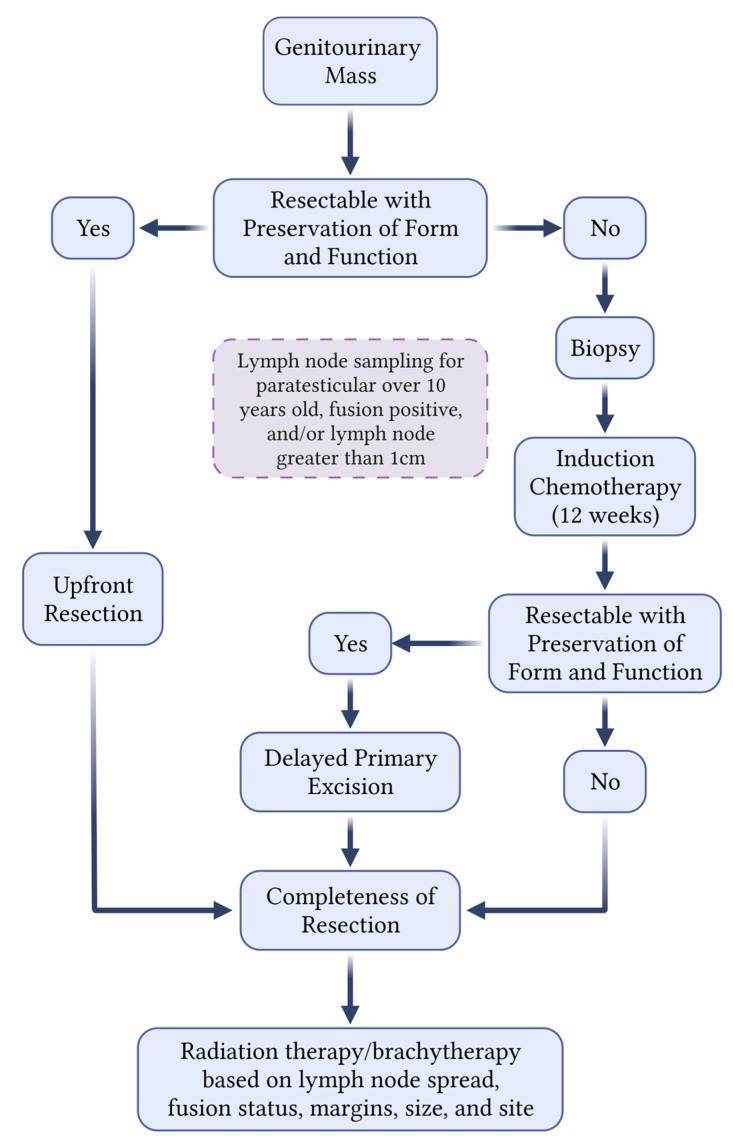
A generalized approach for determining local control strategies for pediatric genitourinary masses concerning for rhabdomyosarcoma.

The three main forms of treatment for RMSs include surgical resection, chemotherapy, and radiation therapy. In general, upfront surgical resection for RMSs is preferential if the tumor is localized and complete resection can be achieved without causing substantial functional or cosmetic defects (Figure 1). Primary surgical resection is less common in the GU tract due to the risk of organ dysfunction and inaccuracy of intraoperative margins [19]. In the majority of FGU-RMSs and BP-RMSs, local surgical control is commonly deferred until after induction therapy but before radiation therapy since the dosage of radiation can be decreased depending on the completeness of surgical resection, with regimens varying based on risk stratification [20]. Upfront surgery is more commonly used for GU RMSs with paratesticular RMSs and, in rare instances, renal RMSs. The principal considerations for GU RMSs will be described in the following sections based on the primary site. These strategies are nuanced, multidisciplinary, and evolving, highlighting the need for pediatric oncology, surgery, urology, and radiation oncology expertise to optimally care for these children [21].

## 7. Bladder/Prostate

### 7.1. Presentation

BP-RMSs and other pelvic RMS tumors are often grouped together due to difficulty in distinguishing the site of origin between the intimately adjacent organs [22]. BP-RMSs may cause genitourinary symptoms, including urinary obstruction, hematuria, and dysuria [23]. Consistent with RMSs originating from other locations, BP-RMSs may also be asymptomatic or cause symptoms outside of the GU tract secondary to mass effect (e.g., constipation) [24]. Males more commonly develop BP-RMSs (2:1) even after attempting to exclude prostate tumors. This predilection may actually reflect challenges with the localization of prostate RMSs. About 75% of BP-RMS patients are less than five years old, with only 13% of patients presenting at over ten years old [25]. Despite 90% of BP-RMSs having embryonal histology and being *PAX/FOXO* fusion-negative, they are generally considered to be an unfavorable location with worse prognosis due to the challenges of achieving complete resection and the decreased use of adjuvant therapy with RT due to concerns over complications related to pelvic radiation [3,25].

### 7.2. Diagnosis

Given the non-specific presenting symptoms of GU-RMSs, there may be an extensive diagnostic work-up prior to identifying an RMS. Of note, there are no lab tests specific to RMSs. Pertinent to BP-RMSs, hematuria in children can be caused by urinary tract infections, vasculopathies, nephropathies, coagulopathies, traumatic injury, tumors, and more. These can be further explored with a renal ultrasound and lab tests (e.g., urine analysis with microscopy, urine electrolytes, 24-h urine collection, basic metabolic panel, serum electrolytes, and serum antinuclear antibodies) [26]. Many of the above etiologies can also cause dysuria which is worked up similarly. Urinary obstruction or acute urinary retention may be caused by urinary tract infections, constipation, calculi, and posterior urethral valves. In addition to the above tests, a bladder ultrasound and urodynamic studies (e.g., uroflowmetry, voiding cytogram, etc.) can be used to further elucidate the cause and location of obstruction [27].

When a mass is identified, the initial steps include defining the size, invasion of adjacent structures, nodal status, and evaluation for metastatic disease. Ultrasound, computed tomography (CT), and magnetic resonance imaging (MRI) have all got uses in the work-up for local and metastatic disease. For all GU RMSs, an MRI with conventional and advanced multiparametric sequences is helpful in distinguishing RMSs in any location from the surrounding soft tissue and, like ultrasound, is favored in pediatric patients to avoid exposure to radiation. However, CT of the chest, abdomen, and pelvis is typically recommended for evaluation of metastatic disease [28,29]. 18F-fluorodeoxyglucose with a CT (FDG-PET-CT) is controversial in the work-up of RMSs due to its low sensitivity and specificity in accurately identifying nodal disease, although it is better than standard imaging techniques based on nodal size [28].

RMSs can have variable appearances on imaging, highlighting the need to obtain a tissue diagnosis [29]. Given the risk of significant urinary dysfunction and difficulty in obtaining negative margins, primary excision is seldom indicated for BP-RMSs [19]. It is imperative to obtain enough tissue from nonnecrotic tumors for an accurate diagnosis and genetic characterization [30]. BP-RMSs can be biopsied endoscopically but may require specialized techniques to obtain large enough samples for a diagnosis [31]. Large image-guided core biopsy and incisional biopsy are also considerations. Poorly executed biopsies and resections can complicate subsequent local therapy strategies and lead to suboptimal outcomes [32].

### 7.3. Treatment

Upfront resection is seldom indicated for BP-RMSs. Treatment algorithms typically define resectable disease as those tumors confined to the dome of the bladder and following which the bladder maintains at least two-thirds of its original capacity [22]. Providers should be cautioned against attempting initial resection if the entire tumor cannot confidently be excised due to high rates of incomplete resection [25]. More often, patients with BP-RMSs are treated with neoadjuvant chemotherapy followed by surgical resection. Bladder preservation has been reported in up to 80% of cases, but only 40% retain normal bladder function [29]. More often, patients with BP-RMSs are treated with neoadjuvant chemotherapy followed by surgical resection. Radiation therapy can also be used, but depending on the protocol may be used prior to or after surgical resection. Brachytherapy is growing in popularity for prostate RMSs as it delivers radiation to a smaller area than conventional external beam radiation therapy. Brachytherapy utilizes implanted internal radiation seeds, wires, or ribbons to reduce secondary functional deficits (e.g., bladder dysfunction, bowel injury, pelvic bone deformities, etc.) [33,34]. Brachytherapy has been shown to improve five-year event-free survival when used with surgery and neoadjuvant chemotherapy but may still cause local toxicity with subsequent bladder and erectile dysfunction [33].

### 7.4. Outcomes

A BP-RMS is considered an unfavorable primary site with over twice the risk of mortality at five years when compared to favorable locations [3]. Patients with BP-RMSs have a five-year overall survival of 84%, but this varies drastically with the extent and type of disease. Invasive embryonal BP-RMS patients have a five-year overall survival of 69%, which declines further for patients with non-embryonal BP-RMSs, where the five-year overall survival is only 47% [25]. The majority of disease recurrences or relapses occur in the first three years following treatment [25]. For patients with BP-RMSs, 15–25% will have disease recurrence or relapse at five years [25,35,36].

Urinary functional outcomes are an important aspect of the management of patients with BP-RMSs. Treatment of BP-RMSs can result in significant bladder dysfunction in 26–40% of patients [37,38]. Even with bladder-sparing therapies, patients are still at risk of bladder dysfunction, urethral stenosis, and urinary tract obstruction [37]. While radiation therapy can aid in local disease control, it can contribute to worse bladder and erectile dysfunction, an unfortunate side effect of radiation focused on the pelvis [33]. Following a radical cystectomy, urinary flow can be managed with incontinent diversion (i.e., ileal or colonic conduits), continent diversion (i.e., anal and urethral), orthotopic neobladder, and many more innovative reconstructive techniques that are in development [39,40]. While functional, these methods have associated limitations and complications, including ureteral reflux, ascending infections, and kidney damage [39].

## 8. Paratesticular

### 8.1. Presentation

Unlike BP-RMSs, paratesticular RMSs are typically identified as an asymptomatic or painful mass in the scrotum [23]. Like other RMSs, paratesticular RMSs are most commonly embryonal but may be of alveolar or spindle-cell histology [7,41]. Patients with paratesticular RMSs typically present before age ten years old; however, some alveolar and spindle-cell subtypes present in the 15–18 year age group [41,42,43].

### 8.2. Diagnosis

For patients presenting with a testicular mass, a high-resolution ultrasound with Doppler evaluation is standard for the initial evaluation. A scrotal MRI may be beneficial for the localization and tumor characterization, which can aid in the diagnosis [44]. It is rare to obtain histologic confirmation prior to resection since pre-operative imaging is usually suggestive of a paratesticular tumor. However, if the biopsy is performed for histologic confirmation, it should be done through the inguinal canal, not the scrotum. This biopsy route potentially avoids disease dissemination, as testicular lymphatics drain to the retroperitoneum, while scrotal lymphatics drain to superficial inguinal lymph nodes [45,46]. All patients should undergo cross-sectional evaluation of the retroperitoneum via an MRI, CT, or FDG-PET-CT. Abnormal lymphadenopathy (>1 cm) noted on cross-sectional imaging or high-risk clinical features (age > ten years of age) merit ipsilateral infra-renal retroperitoneal lymph node sampling of 10–12 lymph nodes [47].

### 8.3. Treatment

Radical orchiectomy is the mainstay of local therapy for paratesticular RMSs. Resection of the tunica vaginalis with the tumor, testis, and spermatic cord up to the level of the internal ring is the standard of care treatment for these RMS tumors [29]. En-bloc scrotal resection is indicated as evidence of direct scrotal invasion at resection. However, resection of the scrotum is no longer recommended for scrotal violation during biopsy or resection. Hemiscrotectomy was historically recommended for patients with scrotal violation during biopsy or resection, but subsequent studies do not demonstrate survival benefits [29,31,48,49].

Surgical lymph node evaluation is performed for appropriate staging in high-risk patients with paratesticular RMSs, given the poor reliability of imaging to identify nodal disease. The lymphatic drainage of the testicles is primarily to the retroperitoneum inferior to the renal vasculature. Retroperitoneal lymph node template dissection was previously recommended for everyone but carries significant morbidity (5–20%), including infertility, bowel obstruction, chyle leak, hydronephrosis, and sexual dysfunction. Currently, ipsilateral retroperitoneal lymph node sampling is only recommended for patients at high risk of regional spread, including patients greater than ten years of age (40% have lymph node involvement) or with concerning imaging findings (>1 cm in diameter) [31,49,50]. Retroperitoneal lymph node sampling focuses on the nodal basins inferior to the ipsilateral renal vein extending laterally to the ureter, inferiorly to the common iliac artery bifurcation, and to the diaphragmatic crura superiorly. A sampling of 7–12 nodes within this area is recommended to identify disease presence/staging [46]. Surgical boundaries should be marked with clips for the potential need for future radiotherapy.

### 8.4. Outcomes

Unlike BP-RMSs, paratesticular RMSs are considered a favorable location with a better overall prognosis [50]. Overall survival for these patients is around 94–99%. Across different treatment regimens, patients ten years or older and/or with tumors larger than 5 cm have consistently worse event-free and overall survival [41,49,51]. Patients treated in the IRS-IV study had a three-year failure-free survival of 90% for patients less than ten years old vs. 63% for older patients [49]. A report from the European pediatric soft tissue sarcoma study group (ESSG) report, based on their 2005 treatment protocol, the event-free survival and five-year overall survival in children less than ten years old was 95.8% and 98.1%, respectively, versus ten years old or older which were 79.6% and 86.7%, respectively [51]. Retroperitoneal lymph node dissection is shown to improve five-year overall survival for patients ten years or older from 64 to 92% [50,52]. Tumors greater than 5 cm have worse outcomes, irrespective of patient age [40]. The average time to recurrence is 13 months, with the majority of recurrences occurring locoregionally [41,51]. Mortality following recurrence is high, with studies reporting a 40–78% mortality rate, highlighting the importance of appropriate and complete initial treatment [41,49].

## 9. Vagina/Vulva/Uterus

### 9.1. Presentation

FGU-RMSs arise from the vulva, vagina, cervix, and uterus. Tumors can present as large protruding masses, vaginal bleeding, abdominal pain, or symptoms from mass effect (e.g., urinary incontinence, urinary frequency, and constipation) [23,53,54]. Similar to the other RMSs presented, these typically present in early childhood at two–five years old, with another increase in incidence in the latter adolescent age group [55,56,57]. FGU-RMSs typically have embryonal histology and a predilection for the botryoid subtype compared to other locations [57].

### 9.2. Diagnosis

FGU-RMSs may present with non-specific symptoms, and the work-up includes a bimanual exam with possible vaginoscopy and cystoscopy under anesthesia. An MRI of the abdomen and pelvis is commonly used to characterize locoregional spread/invasion [58,59]. Once a mass is identified on clinical exam or imaging, tissue must be obtained to confirm the diagnosis histologically. According to a consensus from the COG, ESSG, and the Cooperative Weichteilsarkom Studiengruppe, tumors originating in the vagina and uterus, representing the vast majority of FGU-RMSs, should be biopsied initially. Primary excision can be considered for small exophytic lesions where there are no anticipated functional or cosmetic derangements [59]. For RMSs in the vulva, partial vulvectomy may be required to obtain tissue for diagnosis [60]. Lymphatic spread is rare in FGU-RMSs, and routine lymphadenectomy is not recommended. Suspicious lymph nodes on the imaging evaluation of an exam should be investigated with a biopsy.

### 9.3. Treatment

Historically, FGU-RMSs were treated with aggressive upfront surgical resection, including pelvic exenteration, hysterectomy, and vaginectomy. However, FGU-RMSs generally respond well to chemotherapy which has led to upfront resection falling out of favor to minimize morbidity and preserve excellent outcomes. The focus of the initial surgical treatment in the majority of cases should be staging and biopsy. Upfront surgical resection can be selectively pursued in a minority of FGU-RMS cases where a small, well-circumscribed tumor is present that is amenable to resection with organ preservation and avoids functional or cosmetic deformity. For localized FGU-RMSs treated with upfront biopsies, radiographic and endoscopic surveillance is pursued after induction chemotherapy to assess for clinical response. Tumors that incompletely respond to induction chemotherapy, surgery, or radiation can be considered for local control measures [58]. Brachytherapy is a potential alternative to external beam radiation, featuring a smaller target area and resulting in less morbidity [61,62]. Radiation therapy is recommended in Group II and III FGU-RMSs due to evidence of high rates of recurrence in these patients not treated with radiation therapy [63,64]. In particular, brachytherapy has been shown to be beneficial in the treatment of FGU-RMSs, with local control achieved in 93% of patients [62].

### 9.4. Outcomes

FGU-RMSs are also considered a favorable anatomic location with a good prognosis. These tumors typically have favorable outcomes with upwards of 90% ten-year overall survival rate [57,59,62]. However, survival differs based on patient age, whereby for those younger than 12 months old, the ten-year survival is only 81% [57]. In general, infants with RMSs have been shown to have worse outcome measures. Patients younger than 12 months of age also have higher rates of radiation protocol nonadherence [63]. This is generally thought to be due to provider concerns about the long-term morbidity of radiation in the developing infant.

Recurrence of FGU-RMSs typically occurs within three years following diagnosis [57]. Radiation therapy has been shown to greatly affect the rate of recurrence in FGU-RMSs, which has led to the emphasis on multimodal initial therapy [59]. Overall, survival following recurrence has improved due to aggressive salvage therapy, including surgery and radiation therapy [57,62].

## 10. Kidney

### 10.1. Presentation

An RMS originating in the kidney is very rare, comprising only 0.17% of RMS patients. A review of all patients from the IRS experience, 1972–2005 identified only six patients with renal primary RMSs [65]. These patients can present with abdominal distension, palpable abdominal mass, and hematuria [66,67]. There is a case report of more systemic symptoms (e.g., palpitations and shortness of breath) due to the extension of the tumor into the inferior vena cava [66].

### 10.2. Diagnosis

The differential for a renal mass in childhood includes Wilms tumors, clear cell carcinoma, renal cell carcinoma, rhabdoid tumor, lymphoma, angiomyolipoma, mesoblastic nephroma, and more, which are all more common than renal RMSs [68,69]. Renal RMSs can be difficult to distinguish from Wilms tumors and, historically, have been argued to be a subvariant of the latter [67]. The work-up for childhood renal masses includes urine analyses, blood tests, and renal ultrasound. Abdominal CT scans and MRIs can help distinguish the extent of the tumor as well as any metastatic disease burden but are not adequate at distinguishing between renal RMSs and the other forms of renal tumors [69]. While tissue is necessary for histologic confirmation of disease, a biopsy of renal tumors should be conducted with extreme caution and thoughtful consideration, as a biopsy of some tumors can result in upstaging requiring more extensive treatment [68]. Such decisions regarding biopsy versus upfront resection for histologic confirmation of disease should be made by a multidisciplinary team familiar with the management of the different tumor possibilities [70].

### 10.3. Treatment

Although these tumors have an increased propensity to contain anaplasia, they have a good response to therapy and therefore, tumors amenable to resection are recommended to undergo upfront complete resection with lymph node dissection. Given the infrequent nature of this tumor, it is difficult to draw conclusions, but the recommendation is to treat these patients in protocols designed to treat RMSs from other unfavorable locations with similar histology [65].

### 10.4. Outcomes

Given the rarity of renal RMSs, disease survival is difficult to determine. While the initial response to the therapeutic regimens in the IRS groups was optimal, two of the six patients developed recurrence within a year following therapy, resulting in death by another year. However, these two patients are the only two who had metastatic disease at the time of initial treatment [64]. Subsequently, published case reports have shown similar results, with good prognosis in localized disease [71,72].

## 11. Residual Mass

After the completion of treatment, it is common to have a residual mass. These can represent a residual tumor, scar tissue, or a lesion containing only mature rhabdomyoblasts. Rhabdomyoblasts have been shown to occur as a positive response to chemotherapy [73]. Mature rhabdomyoblasts in BP-RMSs are not indicative of future recurrence nor worse overall survival in RMSs [31,35].

For clinical Group III patients across all primary sites with a residual mass after adjuvant treatment, surgical resection was not associated with improved outcomes or event-free survival [35]. As such, it is not generally recommended to pursue radical resections or escalation of medical therapy for the presence of a residual mass unless the mass is evolving over time or is causing significant symptoms [31,74].

## 12. Long-Term Prognosis

Overall, pediatric patients with RMSs who survive five years from the time of diagnosis have a 90% 15-year survival rate [1]. Ninety-five percent of relapses or recurrences occur within the first three years after diagnosis, and relapse and tumor spread are the biggest contributors to early post-treatment mortality [75,76]. However, these children are treated with additional therapy, which is rarely effective. A major concern in GU tract RMSs is bladder dysfunction occurring as a sequela of surgery, chemotherapy, and/or radiation treatments in the pelvis, as discussed earlier. Females treated for pelvic RMSs have an increased risk of infertility, bladder dysfunction, fistulas, hormone deficiencies, and intestinal strictures, which all increase in the frequency of their occurrence and severity if patients receive pelvic radiation [77]. Patients who underwent extensive retroperitoneal lymph node dissections, particularly for paratesticular RMSs, are at risk of sexual dysfunction and infertility [46].

Although additional chemotherapeutic agents have been suggested, the gold standard therapy is vincristine, dactinomycin, and cyclophosphamide [21]. However, these drugs can have significant side effects. Twenty percent of patients treated in childhood with vincristine have lasting functional deficits (sensory and/or motor) in adulthood from neuropathic injury [78]. This can leave patients in chronic pain and with all the sequelae of trying to manage chronic pain [79]. Dactinomycin has minimal known long-term effects but has been suggested to exacerbate radiation toxicity [80]. Doxorubicin has a well-established risk of cardiotoxicity, which has been reduced with decreased dose regimens. However, adult survivors with childhood exposure to doxorubicin still have an increased risk of developing heart failure with a 50% mortality later in life [81].

Alarmingly, 4% of pediatric cancer survivors will develop a secondary malignancy in their lifetime, which carries a 38% mortality rate [82]. Patients with RMSs are at an increased risk of secondary hematologic malignancies, with an increased risk in patients who underwent stem cell transplants as part of their treatment (a treatment that is not typically employed for RMSs today) [83]. Radiation exposure to different areas of the body increases the risk of cancer in those anatomic locations [82]. Certain chemotherapeutics, such as cyclophosphamide, are also associated with an increased risk of secondary malignancy [82]. Secondary malignancies are a leading late cause of death for childhood survivors and require astute surveillance with an emphasis on early diagnosis [82].

An important facet of care is the lasting psychological effects caused by having childhood cancer and enduring the treatment. Adult survivors of childhood cancer are less likely to be fully employed with a resultant lower income, less likely to graduate college, and less likely to marry/cohabitate. This is likely multifactorial, but adult survivors are more likely to have post-traumatic stress syndrome and suicidal ideation [84,85]. The support these patients require following cancer treatment extends beyond physical health, with an increasing emphasis on mental wellness.

## 13. Future Directions

The treatment of RMSs, and in particular GU RMSs, is continuously evolving with an improved understanding of their clinical courses and delineation of meaningful risk factors. However, these tumors remain a difficult clinical entity to treat given the proximity of other organs with an associated high risk of life-altering side effects. As such, there is an ongoing need to study these tumors to find innovative and effective treatments, as well as determine which tumors are appropriate for treatment de-escalation. Recently, Abbou et al. reported the utility of measuring circulating tumor DNA (ctDNA) as a prognostic marker for intermediate-risk RMSs [86]. Accurate prognostication allows for both appropriately aggressive therapy when needed and therapy de-escalation for others. There are multiple clinical trials focused on improving outcomes, such as prolonging survival and reducing side effects, by optimizing combination therapeutic options. Recent promising agents include temsirolimus, temozolomide, tirapazamine, and long-term maintenance therapy using trofosfamide with etoposide or idarubicin for the treatment of intermediate- and high-risk RMSs [87,88,89,90,91]. Alternatively, other trials are investigating optimizing the delivery of currently used chemotherapeutics, such as using hyperthermic intraperitoneal chemotherapy (HIPEC) with cytoreductive surgery [92]. Ultimately, there is a multitude of clinical trials specific to RMSs, or pediatric solid tumors, allowing for the inclusion of RMSs, which are primed to escalate the care of pediatric RMS patients.

## 14. Conclusions

Children afflicted with RMSs have benefitted from multidisciplinary paradigms utilizing surgical, chemotherapeutic, and radiation therapies. Risk stratification protocols featuring the recent adaptation of molecular features are improving our ability to identify patients in need of more aggressive and novel therapeutic strategies. Another key focus has been identifying patients with favorable prognoses whose treatment can be deescalated to minimize morbidity. Neoadjuvant chemotherapy is being increasingly utilized for BP-RMSs, followed by local control strategies with bladder-preserving surgery and radiation therapy, including brachytherapy. A similar paradigm is emerging in FGU-RMSs with the goal of minimizing functional and cosmetic deformity while preserving excellent survival rates. Surgery remains a mainstay for the treatment of paratesticular RMSs. Children with high-risk diseases still have poor survival predictions despite our most aggressive treatments, highlighting the need to continue to identify new treatment strategies.

## Figures and Tables

**Table 1:** Risk group stratification for RMS. Table adapted from Haduong et al. [11].

Risk Group	Fusion Status/ Molecular Profile	Stage	Group
**Very Low Risk**	Fusion −, *MYOD1* WT, *TP53* WT	1	I
**Low Risk**	Fusion −, *MYOD1* WT, *TP53* WT	1	II, III (orbit)
		2	I, II
**Intermediate Risk**	Fusion −	1	III (non-orbit)
		2	III
		3	I, II, III
		4	IV (age < 10 y)
	Fusion +	1,2,3	I, II, III
**High Risk**	Fusion −	4	IV (age ≥ 10 y)
	Fusion +	4	IV

Fusion status: negative (−) or positive (+) for any FOXO1 fusion. WT: wildtype.
